# 

**Published:** 1952-07

**Authors:** 


					THE BRISTOL
MEDICO-CHIRURGICAL
JOURNAL
VOL. 69. No. 2?i Price 4/-

				

